# Evaluation of the Association between Oral Lichen Planus and Hypothyroidism: a Retrospective Comparative Study

**Published:** 2016-03

**Authors:** Fatemeh Lavaee, Marjan Majd

**Affiliations:** 1 Dept. of Oral & Maxillofacial Disease, School of Dentistry, Shiraz University of Medical Sciences, Shiraz, Iran.; 2 Dept. of Endodontics, School of Dentistry, Kurdistan University of Medical Sciences, Sanandaj, Iran.

**Keywords:** Hypothyroidism, Oral lichen planus, Association, Hashimoto disease, Levothyroxine

## Abstract

**Statement of the Problem:**

Oral Lichen planus (OLP) is an autoimmune mucocutaneous disease. There are some reports of thyroid diseases, especially hypothyroidism, to have association with OLP in some studies.

**Purpose:**

Based on the controversial results of former studies in other populations about the association of hypothyroidism and OLP, the current study aimed to evaluate this association in a sample of Iranian population.

**Materials and Method:**

This retrospective comparative study evaluated 523 patients with OLP referring to the Oral and Maxillofacial Department of Shiraz Dental Faculty as the test group and 523 age- and sex-matched patients as the control group. Those participants with oral lichenoid reactions and other mucosal lesions were excluded. The odds ratio (OR) with 95% confidence intervals (CI) for the association of OLP and thyroid diseases were estimated by logistic regression adjusted for the matched age and sex.

**Results:**

In the test group, 74% (n=387) and in the control group 73.8% of the patients were female (n=386). In the test group, 26% (n=136) and in the control group 26.2% of the samples were male (n=137). A total of 4% of the patients in the control group (n=21) and 6.7% in the case group (n=35) had a history of hypothyroidism. The reported OR for association of thyroid disease and OLP was 1.714 (CI=0.984-2.987).

**Conclusion:**

The results of this study showed no significant association between hypothyroidism and OLP in comparison with the age- and sex-matched control group.

## Introduction

Oral Lichen planus (OLP) is an autoimmune mucocutaneous disease which is more common in females in the fifth decade of their lives. It may have the clinical presentations of both red and white lesions. The main cause of OLP is still unknown; whereas, a complex series of immune-modulated events is responsible for the pathogenesis of OLP. Auto reactive T-lymphocytes are supposed to be the most important cause of OLP.[1]

Chronic oral lesions can relapse several times. Their signs and symptoms vary from asymptomatic to debilitating. There are other allergic reactions with similar clinical or histopathological manifestations named lichenoid reaction. The lichenoid reaction is in relation to some special medications or contact of oral mucosa to some allergic restorations such as amalgam.[1-2] The classification of OLP is based on clinical manifestations including popular, reticular, plaque type, atrophic (erythematous), erosive, ulcerative, and bullous.[3-4]

In some populations, OLP has been reported to be associated with some systemic diseases such as dyslipidemia, hepatitis C, and thyroid disease (particularly hypothyroidism); which are called for further investigations.[5-6] There is a report about the higher levels of antinuclear antibody (ANA), antithyroglobulin (TGA), and antithyroid microsomal antibody in the population of patients with OLP.[7]

Studies reported a connection between thyroid diseases, particularly hypothyroidism, and OLP.[5, 8-9] On the other hand, Compilato *et al.* detected no significant association between the autoimmune thyroid diseases and OLP.[10] Considering the controversial results of former studies in other populations about the association between hypothyroidism and OLP, the current study aimed to evaluate this association in a sample of Iranian population.

## Materials and Method

This retrospective comparative study evaluated 523 patients with OLP referred to the Oral and Maxillofacial Department at Shiraz Dental Faculty, during 2001 to 2014. After evaluation the patients’ records, all the patients who had OLP with clinical impression or histopathological confirmation were included in the case group of this study. The patients were examined by an oral medicine specialist. The patients with reticular, popular, and plaque types of OLP were grouped as keratotic type in this study; other types included atrophic (erythematous), erosive, ulcerative and bullous. Clinical examination of reticular and popular types of OLP and histopathological confirmation for plaque type, atrophic, erosive and ulcerative OLP were used to determine the inclusion criteria for recruiting the patients in the study group. 

Those with oral lichenoid reactions were excluded because of the allergic base of these lesions. The patients with clinical impression of lichenoid reactions (drug-induced or contact reaction), and those with the history of a suspicious medication intake which had induced lichenoid reaction, were considered as drug-induced lichenoid reaction. The lesions with limited extension and also those lesions which were in close contact to allergic materials such as amalgam were diagnosed as contact reaction. The patients with other mucosal lesions were also excluded.

From the patient records of whom visited for routine dental care, 523 age- and sex-matched patients with no oral lesion were selected as the control group; having no more than 5 year difference in age with study group. Selected information including age, sex, type of OLP, any systemic disease especially thyroid disease; the medications used by patients, other oral mucosal diseases excluding the OLP were extracted from the patients' file folder. The thyroid diseases were registered based on the patients’ self-explaining history of systemic diseases and medication.

 OLP patients were classified according to the clinical manifestation as keratotic, erosive, atrophic, ulcerative and bullous. If more than one type of OLP were combined, the most severe type was ultimately chosen as the clinical type of that patient. The odds ratio (OR) with 95% confidence intervals (CI) and Chi-square test were used to evaluate the association between the OLP and thyroid disease. 

## Results

Female patients comprised 74% of the test group (n=387) and 73.8% of the controls (n=386). Male patients constituted 26.2% of the test group (n=136) and 26.2% of the controls (n=137). The mean age of patients in the case and control group was 48.43±13.82 and 48.49±13.33 respectively. No statistically significant difference was detected between the age and sex in case and control group. 

A history of hypothyroidism was observed in 4% of the patients in control (n=21) and 6.7% of the case group 6.7% (n=35). Hypothyroidism was the only thyroid disease mentioned in the history of patients with OLP. The prevalence of thyroid disease (hypothyroidism) was 2.19% among men and 6.46% among women, with an overall prevalence of 5.35% (Table 1).

**Table 1:** The prevalence of hypothyroidism in different sexes

		**Thyroid disease**		**Total**
		**Without** **hypothyroidism**	**With** **hypothyroidism**	
Sex	Males	267(97.81%)	6(2.19%)	273
	Females	723(93.54%)	50(6.46%)	773
Total		990(94.65%)	56(5.35%)	1046

In the case group, the patients who had several types of oral lichen planus, the most severe type was chosen as the clinical type of the lesion. 

Table 2 shows the prevalence of different types of oral lichen planus. The most common type of OLP was atrophic followed by ulcerative, keratotic, and erosive. Hypothyroidism was the most prevalent in patients with keratotic, ulcerative, atrophic, and erosive OLP, respectively. The findings revealed no association between hypothyroidism and any type of OLP (Table 2).

**Table 2:** The relation of each type of OLP with hypothyroidism

**Type of OLP**	**Hypothyroidism**		**Total**	**Fisher's exact test ** **(exact sig-2 sided)**	**OR** **95% CI**
	**Without hypothyroidism**	**With hypothyroidism**			
Other types of OLP Atrophic OLP	287 201	25 10	312 211	0.157	0.571 0.268-1.215
Other types of OLP Keratotic OLP	394 94	25 10	419 104	0.19	1.677 0.779-3.611
Other types of OLP Ulcerative OLP	388 100	24 11	412 111	0.136	1.778 0.843-3.752
Other types of OLP Erosive OLP	395 93	31 4	426 97	0.368	0.548 0.189-1.591
Total patients with OLP	488	35	523		

The overall OR for association between thyroid disease and OLP was 1.714 (95% CI 0.984-2.987). Although the risk of hypothyroidism in the patients with OLP was 1.74 times more than normal population, Chi-square test showed no significant association between hypothyroidism and incidence of OLP. 

## Discussion

According to the results of this study, no significant association was detected between hypothyroidism and OLP; nor was it observed between hypothyroidism and any type of OLP in comparison with age- and sex-matched control group. 

Previous studies on different populations and geographic areas reported controversial results. Compilato *et al.* observed that OLP had no significant relation to thyroid-related medications and autoimmune diseases,[9] which confirms the results of our study. Both studies omitted oral lichenoid lesions from their evaluations; however, Compilato evaluated the possible association between OLP and Hashimoto thyroiditis and Graves’ disease, as well.[9]

On the other hand in a retrospective case-control study, Siponen *et al.* evaluated the association of any thyroid diseases including hypothyroidism and thyroid-related medications with the incidence of OLP or oral lichenoid lesions. They reported OR of 2.12 (CI 1.06 to 4.21) for the patients with OLP and any thyroid diseases and OR of 2.39 (CI 1.05-5.61) for the patients with OLP and hypothyroidism. The OR was reported to be 1.57 (CI 0.62-3.73) in patients with oral lichenoid lesions and any thyroid diseases, and 1.73 (CI 0.56-4.9) in those with oral lichenoid lesions and hypothyroidism. These results confirmed the relationship between any thyroid disease and OLP, but not with oral lichenoid lesions.[7]

Similar results were achieved in a cross-sectional survey by Lo Muzio et al. in 2013 (*p*< 0.0003, OR= 14.29, 95%CI 1.9-106.2). Considering the significantly higher prevalence and the onset timing of Hashimoto's thyroiditis in patients with OLP, they reported Hashimoto's thyroiditis as a predisposing factor for OLP.[6]

Robledo-Sierra et al. registered the medication of two groups of OLP patients and healthy controls. They reported significantly higher consumption of thyroid medications (Levothyroxine) and NSAIDs (non-steroidal anti-inflammatory drugs) in OLP patients (*p*< 0.01, OR=3.39, 95%CI 0.09-5.46).[10] These results confirmed significant relation between consumption of Levothyroxine and OLP. The same findings were reported by Hirota *et al.* and Kragelund *et al.*[11-12] Hirota also stated that OLP was positively related to hypothyroidism.[11]

Some studies evaluated the serum level of thyroid-related antibodies. Chang *et al.* in 2009 stated that there was a significantly higher serum level of ANA (antinuclear antibody), antigastric parietal cell (GPCA), thyroglobulin auto-antibody (TGA), and antithyroid microsomal autoantibody (TMA) in 320 Chinese patients with OLP in comparison with 53 healthy patients (*p*< 0.005).[8] Some other surveys approved the result of the above-mentioned study. They reported incidence of 27-82% positive autoantibody in OLP and erosive OLP patients.[5, 13-18] These autoantibodies were decreased after using some specific medication such as topical triamcinolone[19] and systemic Levamisole.[16]

Lundstrom reported 27% serum autoantibody positivity including rheumatoid factor (RF), ANA, and anti-smooth muscle autoantibody (SMA) in OLP patients.[13] Besides hypothyroidism and thyroid diseases, a possible association has been also reported between OLP and other immune-mediated disease,[20] namely ulcerative colitis and alopecia areata.[21] Systemic lupus erythematosus and rheumatoid arthritis were reported to be associated with autoimmune thyroid disease.[22]

The concept of possible relation or association between OLP and thyroid diseases has originated from several reports of patients who were affected by both OLP and thyroid diseases. Since there are several evidences about the association of some autoimmune diseases and autoimmune thyroid diseases,[22]researchers have investigated the relationship between OLP as an autoimmune disease and autoimmune thyroid disease. Patients with any kind of autoimmune disease are more susceptible to other types of autoimmune diseases; however, it varies according to many factors such as the prevalence and incidence of that disease.[22] The authors suggest that genetic predisposition of a population and the environmental factors might affect the prevalence and incidence of these diseases. There is no report on the biological relation of these two diseases. The current research could not show any association between OLP and thyroid diseases.

An investigation on a selected Iranian population reported the prevalence of hypothyroidism to be 4.8% and 12.8 in men and women, respectively.[23] The current study assessed the prevalence of thyroid disease to be 2.19% and 6.46% in men and women, respectively. Such a difference can be due to the various methods of sampling. Different ethnicity of studied population might be another justifying reason.

Diversity of races and ethnicities and different ranges of prevalence of autoimmune diseases including hypothyroidism and OLP can be some reasons of the controversial results reported in several countries. The patients with OLL were excluded from the current study; while, in some other studies[6-7] they were not excluded; this difference can play as an interfering factor. Furthermore, different sample sizes were considered in various studies, some of which were not sufficient for exact evaluation of the relation and association. It was better to specify the sample size of test group and healthy controls according to the prevalence of hypothyroidism and OLP in different countries.

Due to the retrospective method of most researches, presence of some incorrect or missing data of patients especially about their medication and presence of thyroid disease was possible. 

Undoubtedly, future researches and more exact information are recommended. Since the types of hypothyroidism of patients recruited in this study were not recorded in their file, it was impossible to precisely differentiate the etiological types of hypothyroidism and their association with OLP. Diagnosing the etiology of hypothyroidism, particularly the immune-mediated types, and evaluating their association with OLP separately can yield more precise results regarding the immune-mediated basis of OLP. 

It would be better to examine the presence of thyroid disease through measuring serum level of thyroid hormones and autoantibodies besides the medical history of OLP patients. Additionally, specifying larger sample sizes can reduce the confounding factors. 

## Conclusion

This study observed no association between the hypothyroidism and OLP in the evaluated population. The autoimmune diseases had different incidence rate in different countries. Considering these varieties, controversial results about the association of hypothyroidism and OLP have been reported. 

## References

[1] Au J, Patel D, Campbell JH (2013). Oral lichen planus. Oral Maxillofac Surg Clin North Am.

[2] Thongprasom K, Carrozzo M, Furness S, Lodi G (2011). Interventions for treating oral lichen planus. Cochrane Data base Syst Rev.

[3] Little JW, Falace D, Miller C, Rhodus NL (2013). Dental Management of the Medically Compromised Patient.

[4] Yardimci G, Kutlubay Z, Engin B, Tuzun Y (2014). Precancerous lesions of oral mucosa. World J Clin Cases.

[5] Chang JY, Chiang CP, Hsiao CK, Sun A (2009). Significantly higher frequencies of presence of serum autoantibodies in Chinese patients with oral lichen planus. J Oral Pathol Med.

[6] Lo Muzio L, Santarelli A, Campisi G, Lacaita M, Favia G (2013). Possible link between Hashimoto's thyroiditis and oral lichen planus: a novel association found. Clin Oral Investig.

[7] Siponen M, Huuskonen L, Läärä E, Salo T (2010). Association of oral lichen planus with thyroid disease in a Finnish population: aretrospective case-control study. Oral Surg Oral Med Oral Pathol Oral Radiol Endod.

[8] Chang JY, Chiang CP, Hsiao CK, Sun A (2009). Significantly higher frequencies of presence of serum autoantibodies in Chinese patients with oral lichen planus. J Oral Pathol Med.

[9] Compilato D, Paderni C, Di Fede O, Gulotta G, Campisi G (2011). Association of oral lichen planus with thyroid disease in a Finnish population: a retrospective case-control study: "A different finding from a Mediterranean area". Oral Surg Oral Med Oral Pathol Oral Radiol Endod.

[10] Robledo-Sierra J, Mattsson U, Jontell M (2013). Use of systemic medication in patients with oral lichen planus - a possible association with hypothyroidism. Oral Dis.

[11] Hirota SK, Moreno RA, dos Santos CH, Seo J, Migliari DA (2011). Analysis of a possible association between oral lichen planus and drug intake. A controlled study. Med Oral Patol Oral Cir Bu-cal.

[12] Kragelund C, Thomsen CE, Bardow A, Pedersen AM, Nauntofte B, Reibel J (2003). Oral lichen planus and intake of drugs metabolized by polymorphic cytochrome P450 enzymes. Oral Dis.

[13] Lundström IM (1985). Serum immunoglobulins and autoantibodies in patients with oral lichen planus. Int J Oral Surg.

[14] Carrozzo M, Gandolfo S, Lodi G, Carbone M, Garzino-Demo P, Carbonero C (1999). Oral lichen planus in patients infected or noninfected with hepatitis C virus: the role of autoimmunity. J Oral Pathol Med.

[15] Lin SC, Sun A, Wu YC, Chiang CP (1992). Presence of anti-basal cell antibodies in oral lichen planus. J Am Acad Dermatol.

[16] Sun A, Chiang CP, Chiou PS, Wang JT, Liu BY, Wu YC (1994). Immunomodulation by levamisole in patients with recurrent aphthous ulcers or oral lichen planus. J Oral Pathol Med.

[17] Lodi G, Olsen I, Piattelli A, D'Amico E, Artese L, Porter SR (1997). Antibodies to epithelial components in oral lichen planus (OLP) associated with hepatitis C virus(HCV) infection. J Oral Pathol Med.

[18] Lukac J, Brozović S, Vucicević-Boras V, Mravak-Stipetić M, Malenica B, Kusić Z (2006). Serum autoantibodies to desmogleins 1 and 3 in patients with oral lichen planus. Croat Med J.

[19] Sun A, Chia JS, Chang YF, Chiang CP (2002). Serum interleukin-6 level is a useful marker in evaluating therapeutic effects of levamisole and Chinese medicinal herbs on patients with oral lichen planus. J Oral Pathol Med.

[20] Scully C, Beyli M, Ferreiro MC, Ficarra G, Gill Y, Griffiths M (1998). Update on oral lichen planus: etiopathogenesis and management. Crit Rev Oral Biol Med.

[21] (1991). Epidemiological evidence of the association between lichen planus and two immune-related diseases. Alopecia areata and ulcerative colitis. Gruppo Italiano Studi Epidemiologici in Dermatologia. Arch Dermatol.

[22] Lazúrová I, Benhatchi K, Rovenský J, Kozáková D, Wagnerová H, Tajtáková M (2009). Autoimmune thyroid disease and autoimmune rheumatic disorders: a two-sided analysis. Ann N Y Acad Sci.

[23] Aminorroaya A, Janghorbani M, Amini M, Hovsepian S, Tabatabaei A, Fallah Z (2009). The prevalence of thyroid dysfunction in an iodine-sufficient area in Iran. Arch Iran Med.

